# A Toll-like receptor 2 agonist-fused antigen enhanced antitumor immunity by increasing antigen presentation and the CD8 memory T cells population

**DOI:** 10.18632/oncotarget.9001

**Published:** 2016-04-26

**Authors:** Chiao-Chieh Wu, Shih-Jen Liu, Hsin-Wei Chen, Kuan-Yin Shen, Chih-Hsiang Leng

**Affiliations:** ^1^ Graduate Institute of Life Sciences, National Defense Medical Center, Taipei 114, Taiwan; ^2^ National Institute of Infectious Disease and Vaccinology, National Health Research Institutes, Miaoli County 350, Taiwan; ^3^ Graduate Institute of Immunology, China Medical University, Taichung 40402, Taiwan

**Keywords:** rlipo-immunogen, Toll-like receptor 2, antigen presentation, tumor regression, memory T cells

## Abstract

The induction of long-lived effector CD8^+^ T cells is key to the development of efficient cancer vaccines. In this study, we demonstrated that a Toll-like receptor 2 (TLR2) agonist-fused antigen increased antigen presentation via TLR2 signaling and induced effector memory-like CD8^+^ T cells against cancer after immunization. The N-terminus of ovalbumin (OVA) was biologically fused with a bacterial lipid moiety TLR2 agonist to produce a recombinant lipidated ovalbumin (rlipo-OVA). We demonstrated that rlipo-OVA activated bone marrow-derived dendritic cells (BM-DCs) maturation and increased antigen presentation by major histocompatibility complex (MHC) class I via TLR2. After immunization, rlipo-OVA skewed the immune response towards T helper (Th) 1 and induced OVA-specific cytotoxic T lymphocyte (CTL) responses. Moreover, immunization with rlipo-OVA induced higher numbers of effector memory (CD44^+^CD62L^−^) CD8^+^ T cells compared with recombinant ovalbumin (rOVA) alone or rOVA mixed with the TLR2 agonist Pam3CSK4. Accordingly, the CD27^+^CD43^+^ effector memory CD8^+^ T cells expressed high levels of the long-lived CD127 marker. The administration of rlipo-OVA could inhibit tumor growth, but the anti-tumor effects were lost after the depletion of CD8 or CD127 cells *in vivo*. These findings suggested that the TLR2 agonist-fused antigen induced long-lived memory CD8^+^ T cells for efficient cancer therapy.

## INTRODUCTION

Cancer vaccines are designed to prevent cancer formation or eliminate cancer cells. To eliminate cancer cells, the induction of antigen-specific CD8^+^ T cells with cytolytic activity capable of killing cancer cells is critical [1]. Although many different cancer vaccine approaches have been tested in cancer patients, their therapeutic efficacy is still limited. Recently, the quantity and quality of antigen-specific cytotoxic T lymphocytes (CTLs) have been proposed to play an important role in their therapeutic effects [2]. After priming by antigen-presenting cells (APCs), CD8^+^ T cells present an antigenic peptide on major histocompatibility complex (MHC) class I molecules. Subsequently, the primed antigen-specific CD8^+^ T cells undergo clonal expansion, resulting in the contraction of effector cells and the establishment of memory cells [3]. The induction of long-lived memory cells is considered an important factor in efficient anti-tumor immunity. Ideally, a cancer vaccine should skew the T cell response to T helper (Th) 1 responses that facilitate the production of potent CTLs and memory CD8^+^ T cells [1, 4, 5].

Protein-based immunogens as cancer vaccines have low toxicity and a very good safety profile. However, the immunogenicity of protein immunogens is low, particularly for the induction of CTL responses [6]. Typically, exogenous protein immunogens are taken up and digested into small peptides for presentation by MHC class II molecules on APCs to prime CD4^+^ T cells. Additionally, certain proteins can be processed and cross-presented by the MHC class I pathway for CD8^+^ T cell priming [7, 8]. Different approaches have been reported to increase the cross-presentation of protein immunogens, such as Toll-like receptor (TLR) agonists [9–11]. Moreover, innate receptor agonists have been shown to increase the cross-presentation of exogenous antigens [12–14]. In our previous study, we showed that TLR2 agonist-fused peptides could be cross-presented to CD8^+^ T cells via a Rab7-regulated endosomal pathway [15]. These innate receptor agonists not only activated APCs to express co-stimulatory molecules but also increased antigen presentation. Therefore, the co-delivery of innate receptor agonists and antigens is an important approach to prime CTL responses. Recently, TLRs were also found to be capable of inducing important specialized groups of memory T cells [3]. Memory CD8^+^ T cells can be divided into two major subsets: effector memory T cells (T_EM_) and central memory T cells (T_CM_). In humans, T_EM_ (CD62L^lo^CCR7^lo^) cells are defined by their lack of lymph node-homing molecules that are capable of immediate effector functions; these cells are different from T_CM_ (CD62L^hi^CCR7^hi^) cells that express lymph node-homing molecules and are specialized for the proliferation of effector functions upon reinfection [16, 17]. In mice, CD44 is a conservative marker expressed at high levels on the surface of all memory T cells, irrespective of their activation status [18, 19]. Phenotypic features could also be defined using a homing molecule such as CD62L, resulting in identification of the T_EM_ (CD44^high^CD62L^low^) and T_CM_ (CD44^high^CD62L^high^) cell subsets. However, Hikono et al. reported that both expression of CD27 and CD43 which are required for generating long-term maintenance of T cell immunity and more closely related to their functions on phenotypic memory CD8^+^ T cells, respectively. The CD27^high^CD43^low^ cell subset showed optimal recall proliferation and superior functions [20–24]. Moreover, CD127 expression was use to characterize long-lived memory T cells [25]. Generally, CD8^+^ T cells with a functionally good memory express high levels of CD127 and CD27 and low levels of CD43 [26–28]. Therefore, identifying novel methods to direct the differentiation of antigen-specific CD8^+^ T cells into functional long-lived memory CD8^+^ T cells is critical for efficient cancer immunotherapy. Previously, we established a platform for the high-yield production of recombinant lipoproteins [29, 30] that were TLR2 agonist-fused proteins. The lipid moiety of the recombinant lipoprotein differs from that of synthetic tri-acylated lipopeptides [31]. This distinction enables the recombinant lipoprotein to elicit different immune responses compared with the synthetic lipopeptide, resulting in the induction of different levels of biological cytokines and chemokines [32]. Additionally, we demonstrated that recombinant lipidated human papillomavirus (HPV) E7 induced CTLs and provided potential protective immunity against cervical cancer in a mouse model [33]. In this report, we used ovalbumin (OVA) as a model immunogen to demonstrate that lipidated immunogens can be efficiently cross-presented to CD8^+^ T cells and induce high-quality memory CD8^+^ T cells. Importantly, depletion of CD127^+^ T cells led to the loss of the anti-tumor effects of the recombinant lipoimmunogen. Thus, CD127^+^cells are necessary for anti-tumor immunity.

## RESULTS

### Production and characterization of recombinant ovalbumin (rOVA) and recombinant lipidated ovalbumin (rlipo-OVA)

**Figure 1:**
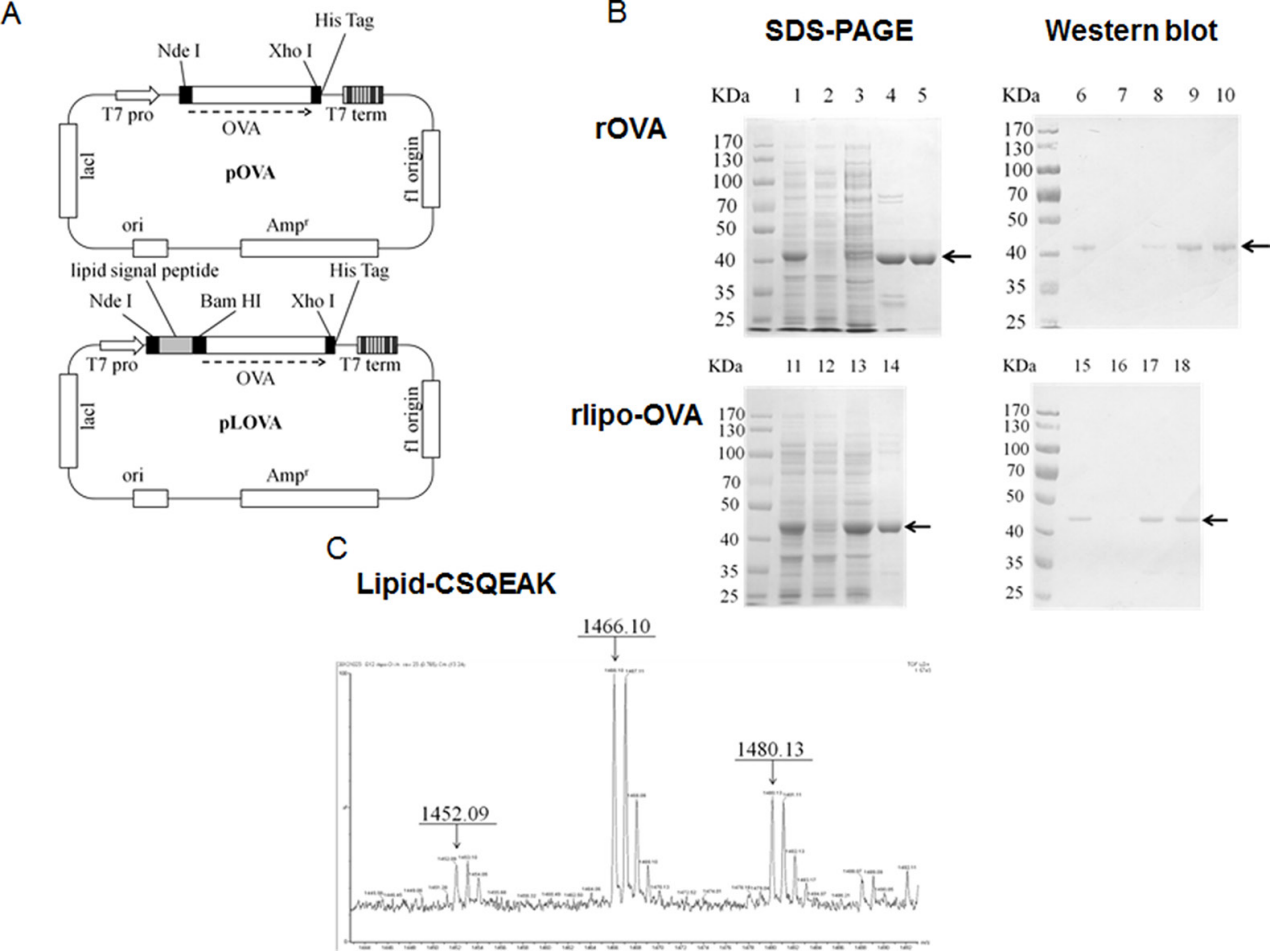
Construction, production and identification of rOVA and rlipo-OVA (**A**) The plasmid maps of pOVA and pLOVA that express rOVA and rlipo-OVA, respectively. (**B**) The rOVA and rlipo-OVA protein purification process used 10% reducing SDS-PAGE followed by Coomassie Blue staining and anti-HisTag antibodies for immunoblotting. The recombinant rOVA was expressed in the *E. coli* strain BL21 (DE3). Lane 1, rOVA expression after IPTG induction; lane 2, protein expression in the absence of IPTG induction; lane 3, rOVA extracted fraction; lane 4, recombinant rOVA purified by Ni-NTA resin; and lane 5, polished recombinant rOVA by Q sepharose resin. Lanes 6–10 show immunoblotting to monitor the process of rOVA purification; these lanes are the same as lanes 1–5, respectively. The recombinant rlipo-OVA was expressed in the *E. coli* strain C43 (DE3). Lane 11, rlipo-OVA expression after IPTG induction; lane 12, protein expression in the absence of IPTG induction; lane 13, rlipo-OVA extracted fraction; and lane 14, rlipo-OVA protein purified by Ni-NTA resin. Lanes 15–18 show immunoblotting to monitor the rlipo-OVA purification process; the samples in these lanes are the same as those in lanes 11–14, respectively. The arrows indicate the electrophoretic positions of rOVA or rlipo-OVA in the SDS gels or blots. (**C**) N-terminal rlipo-OVA fragments were obtained and identified after 3 days of digestion. The digested sample was analyzed on a Waters^R^ MALDI micro MX^™^ mass spectrometer. The MALDI-TOF MS spectra revealed lipid peptide signals with three m/z value peaks of 1452.09, 1466.10, and 1480.13.

Following our previous procedure, the ovalbumin (OVA) gene was cloned into the expression vector with and without the lipid signal peptide to construct the plasmids pLOVA and pOVA for the expression of rlipo-OVA and rOVA, respectively [29]. The recombinant antigens were engineered to contain a hexahistidine tag (HisTag) at their C-terminus and expressed in an *Escherichia coli* system under the control of the T7 promoter (Figure 1A). rOVA was purified from the lysates using immobilized metal affinity chromatography (IMAC) and polished using anion-exchange chromatography (Figure 1B, lanes 1–5). The purified protein was analyzed by immunoblotting with an anti-His tag antibody (Figure 1B, lanes 6–10). rlipo-OVA was purified using IMAC (Figure 1B, lanes 11–14). The recombinant protein was detected with an anti-His tag antibody (Figure 1B, lanes 15–18).

rlipo-OVA and rOVA were digested with trypsin to monitor their peptide mass fingerprinting (PMF) by MALDI-TOF mass spectrometry. The results confirmed that the major peaks in the mass spectra corresponded to m/z values derived from rlipo-OVA and rOVA (data not shown). The identification of the lipid moiety in rlipo-OVA was similar to our previous reports [29, 31]. Briefly, the N-terminal fragments from the digested rlipo-OVA were purified and identified using mass spectrometry. Three peaks with m/z values of 1452, 1466 and 1480 (Figure 1C) corresponded to the lipid-modified CSQEAK sequence. After the lipopolysaccharide (LPS) was removed (less than 0.01 EU/mg), purified rlipo-OVA, rOVA and OVA from egg whites were comparatively analyzed for their immunogenicity and efficacy in animal models.

### Bone marrow-derived dendritic cells (BM-DCs) were activated by rlipo-OVA via TLR2

**Figure 2:**
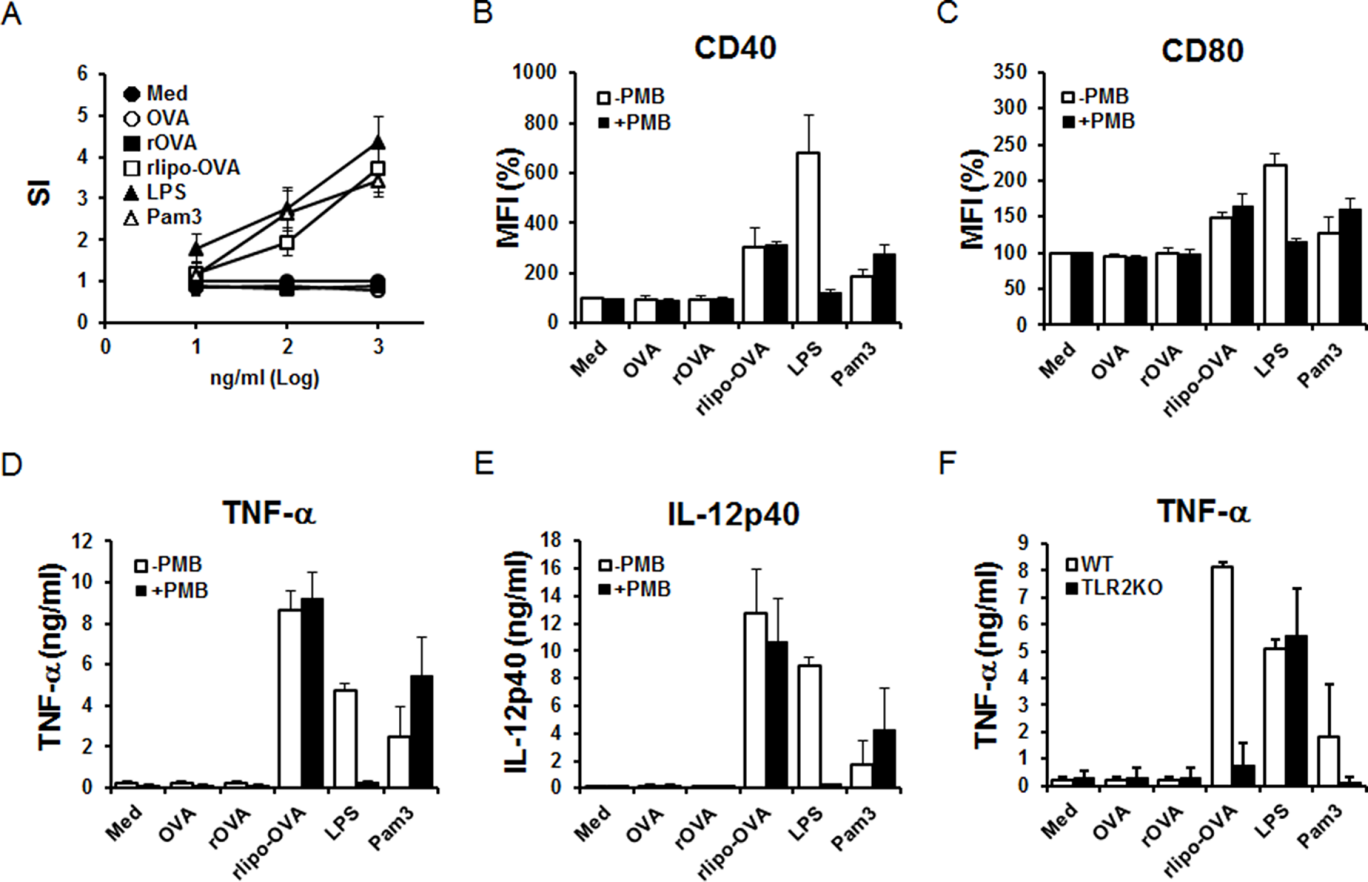
rlipo-OVA stimulates immune cell activation via TLR2 (**A**) Splenocytes were isolated from wild-type C57BL/6 mice and seeded at a density of 2.5 × 10^5^ cells/well in 96-well cell culture plates. The cells were incubated with different concentrations of LPS (10–1000 ng/ml), Pam3 (10–1000 ng/ml), OVA (10–1000 ng/ml), rOVA (10–1000 ng/ml) or rlipo-OVA (10–1000 ng/ml) for 72 h. After 72 h, 10% CellTiter 96^®^ AQ_ueous_ One Solution Reagent was added to each well and the OD_490_ was measured. Stimulation index: (SI) = OD_490_ of the stimulated cells/ OD_490_ of the negative controls. The data represent the mean ± SD of six animals. BM-DCs from wild-type mice were cultured in medium supplemented with LPS (100 ng/ml), Pam3 (100 nM), OVA (2.5 μg/ml), rOVA (2.5 μg/ml) or rlipo-OVA (2.5 μg/ml) in the presence or absence of polymyxin B (50 μg/ml). After an 18-h incubation, the dendritic cells were pre-gated on the CD11c^+^ cell population to measure the expression of the cell surface markers CD40 (**B**) and CD80 (**C**) by flow cytometry. The mean fluorescence intensity (MFI)% for cells cultured in medium was defined as 100%, and the independent experiments were performed in triplicate. For the inflammatory cytokine secretion studies, the supernatants were collected and analyzed for TNF-α (**D**) and IL-12 (p40) (**E**) production by ELISA. Three independent experiments were performed, and the data are presented as the mean + SD. (**F**) BM-DCs from WT or TLR2KO mice were cultured either in medium alone or in medium supplemented with LPS (100 ng/ml), Pam3 (100 nM), OVA (2.5 μg/ml), rOVA (2.5 μg/ml) or rlipo-OVA (2.5 μg/ml). After an 18-h incubation, the supernatants were collected and analyzed for TNF-α production by ELISA. The data are presented as the mean + SD, *n* = 3.

Splenocytes were isolated and stimulated with recombinant immunogens and positive control reagents (LPS and Pam3 are TLR4 and TLR2 agonists, respectively) to determine the proliferative responses. The results showed that rlipo-OVA stimulated the proliferation of splenocytes at concentrations of 10 ng/ml, 100 ng/ml and 1000 ng/ml. In contrast, OVA and rOVA failed to stimulate splenocyte proliferation (Figure 2A). To test their activity on the maturation of dendritic cells, BM-DCs were stimulated with rOVA and rlipo-OVA. The co-stimulatory molecules CD40 and CD80 were up-regulated by rlipo-OVA but not OVA or rOVA (Figure 2B and 2C). The secretion of TNF-α and IL-12p40 from BM-DCs was detected after stimulation with rlipo-OVA but not OVA and rOVA (Figure 2D and 2E). To exclude the effect of residual endotoxin in rlipo-OVA, polymyxin B (PMB) was mixed with the recombinant immunogens to stimulate BM-DCs. Our data showed that there were no significant effects on the stimulatory properties of rlipo-OVA. These results confirmed that the activation of BM-DCs by rlipo-OVA was due to the lipid moiety of rlipo-OVA (Figure 2B–2E).

BM-DCs from wild-type (WT) and TLR2-knockout (TLR2KO) mice were employed to investigate whether rlipo-OVA activated BM-DCs via TLR2. Our results showed that rlipo-OVA and Pam3 stimulated the BM-DCs of WT mice, but not the TLR2KO mice, to secrete TNF-α (Figure 2F). These data demonstrated that rlipo-OVA activated BM-DCs via TLR2 signaling.

### BM-DCs pulsed with rlipo-OVA increased the presentation of OVA-H-2K^b^ via TLR2 signaling

**Figure 3:**
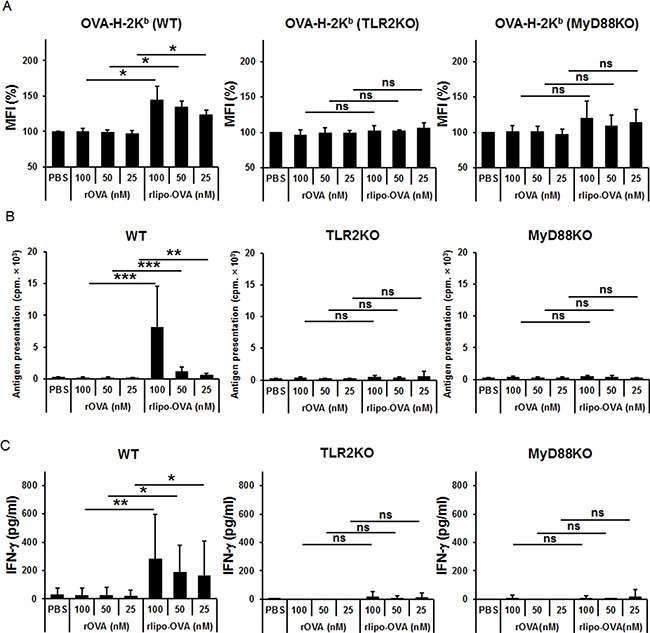
rlipo-OVA increases BM-DCs presenting the OVA-H-2K^b^ molecule via TLR2 signaling (**A**) BM-DCs from WT, TLR2KO and MyD88KO mice were incubated for 24 h with PBS, (100–25 nM) rOVA, or (100–25 nM) rlipo-OVA, and the OVA-H-2K^b^ OVA-peptide were assessed by flow-cytometry analysis. Cells were stained with the PE-labeled 25-D1.16 antibody that recognizes OVA-derived SIINFEKL (SII) assembled with BM-DCs H-2K^b^. (**B**) Antigen presentation was determined by cell proliferation using a [^3^H]thymidine incorporation assay. The antigen-pulsed BM-DCs (1 × 10^4^) were cultured with 1 × 10^5^ CD8^+^ T cells of OT-1 mice for 72 h. Average cpm incorporated in triplicate samples are shown (+SD). (**C**) At 5 day after the antigen-pulsed BM-DCs were cultured with CD8^+^ T cells of OT-1 mice, supernatant was collected and IFN-γ was measured using ELISA. The data are expressed as the means + SD from three independent experiments.

Because a TLR2 agonist-conjugated peptide could be taken up and used to activate CD8^+^ T cells [15], we investigated whether the presentation of peptide/MHC I complexes was indeed increased on the surface of dendritic cells. Peptide/MHC I complexes on antigen-pulsed BM-DCs were analyzed using the 25-D1.16 monoclonal antibody that recognized the SIINFEKL peptide (OVA_257-264_) and MHC class I H-2K^b^ molecule complex (OVA-H-2K^b^). OVA-H-2K^b^ was increased in the rlipo-OVA-pulsed BM-DCs of WT mice but not in the rOVA-pulsed BM-DCs of WT mice. Moreover, the increased presentation of OVA-H-2K^b^ was lost or reduced on rlipo-OVA-pulsed BM-DCs from the TLR2KO and myeloid differentiation primary response gene 88-knockout (MyD88KO) mice (Figure S1). Accordingly, OVA-H-2K^b^ presentation was determined using different doses (25, 50, 100 nM) of rlipo-OVA and rOVA-pulsed BM-DCs from the WT, TLR2KO and MyD88KO mice (Figure 3A). Additionally, the antigen presentation was assessed by T cell activation using [^3^H]thymidine incorporation (Figure 3B) and IFN-γ (Figure 3C). The increased antigen presentation of the rlipo-OVA-pulsed BM-DCs could increase OT-1 cells proliferation and IFN-γ secretion in WT mice but not TLR2KO and MyD88KO mice. These data corresponded with the SII/H-2K^b^ complexes formation that were detected as shown in the Figure 3A. These results directly showed that the lipidated immunogen indeed increased the presentation of peptide/MHC I complexes on the surfaces of dendritic cells.

### OVA-specific T cell responses were elicited by immunization with rlipo-OVA

**Figure 4:**
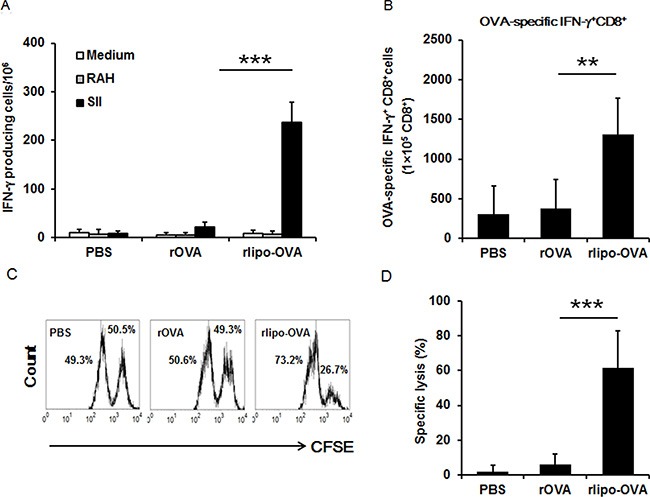
Immunization with rlipo-OVA induces higher levels of OVA-specific cytotoxic T cell responses compared to immunization with rOVA C57BL/6 mice were immunized twice by subcutaneous injection at one-week intervals. The experiments were performed after seven days of last immunization. (**A**) A density of 5 × 10^5^ splenocytes (per well) from immunized mice were incubated with 2.5 μg/ml of the irrelevant RAHYNIVTF (RAH) peptide or SII peptide for 48 h in an anti-IFN-γ-coated 96-well ELISPOT plate. The IFN-γ-secreting spots were calculated using an ELISPOT reader. The data are expressed as the mean + SD, *n* = 6 / per group. (**B**) The SII-specific IFN-γ production in CD8^+^ T cells. Splenocytes were isolated from each group of vaccinated mice. The cells were re-stimulated with 1 μg/ml of the SII peptides for 20 h; for the final 4 h, the cells were treated with 10 ng/ml phorbol 12-myristate 12-acetate (PMA), 1 μg/ml ionomycin, and Brefeldin A solution. The percentages of CD8^+^/IFN-γ^+^ T cells pre-gated on CD3^+^ cells were determined by flow cytometry. The data are presented as the mean + SD, *n* = 5. (**C**) The graphs show the specific lysis of SII peptide-pulsed targets (CFSE^high^) and the un-pulsed peptide control (CFSE^low^). The graph summarizes the results of the specific lysis *in vivo* (**D**). The following equation was used to analyze specific lysis:% Specific lysis = [(% non-peptide × A) −% SII peptide] / (% non-peptide × A). Adjustment factor A = SII peptide/non-peptide from the naïve controls. The data are expressed as the mean + SD of nine animals per group.

C57BL/6 mice were subcutaneously immunized at the base of the tail on days 0 and 7 with rlipo-OVA, rOVA and PBS to determine the OVA-specific CTL responses. The splenocytes of the immunized mice were stimulated with the OVA-specific peptide SIINFEKL (SII, OVA_257-264_) and the irrelevant peptide RAHYNIVTF (RAH, HPV 16 E7_49-57_) to measure the number of OVA-specific IFN-γ-secreting cells using the ELISPOT assay (see Materials and Methods). The results showed that immunization with rlipo-OVA induced higher numbers of SII-specific INF-γ-secreting cells (238.0 ± 41.2) compared with rOVA (22.7 ± 9.8; rOVA vs. rlipo-OVA, *p* < 0.001; Figure 4A). We validated the OVA-specific IFN-γ^+^ CD8^+^ T cells using flow cytometry. The mice immunized with rlipo-OVA showed a significantly higher level of CD8^+^ SII-specific IFN-γ-producing CD8^+^ cells (1316.4 ± 452.0) compared with rOVA (383.8 ± 361.6; rOVA vs. rlipo-OVA, *p* < 0.01; Figure 4B).

To investigate the killing activity of T cells after the administration of rlipo-OVA and rOVA, the immunized mice were adoptively transferred with carboxyfluorescein diacetate succinimidyl ester (CFSE)-labeled and SII-pulsed cells for 18 h. The CFSE-labeled cells could be monitored to evaluate the specific killing ability of the immunized mice. The results demonstrated that rlipo-OVA immunization induced a higher level of OVA-specific CTL activity *in vivo* than rOVA immunization (Figure 4C). The specific lysis were calculated as 2.03 ± 3.51%, 6.23 ± 5.80%, and 61.72 ± 21.25% in the PBS, rOVA and rlipo-OVA immunized mice, respectively (rOVA vs. rlipo-OVA, *p* < 0.001; Figure 4D). These results clearly demonstrated that immunization with the lipidated immunogen induced stronger antigen-specific CTL responses compared with the non-lipidated immunogen.

### rlipo-OVA elicits specific memory CD8^+^ T cells

**Figure 5:**
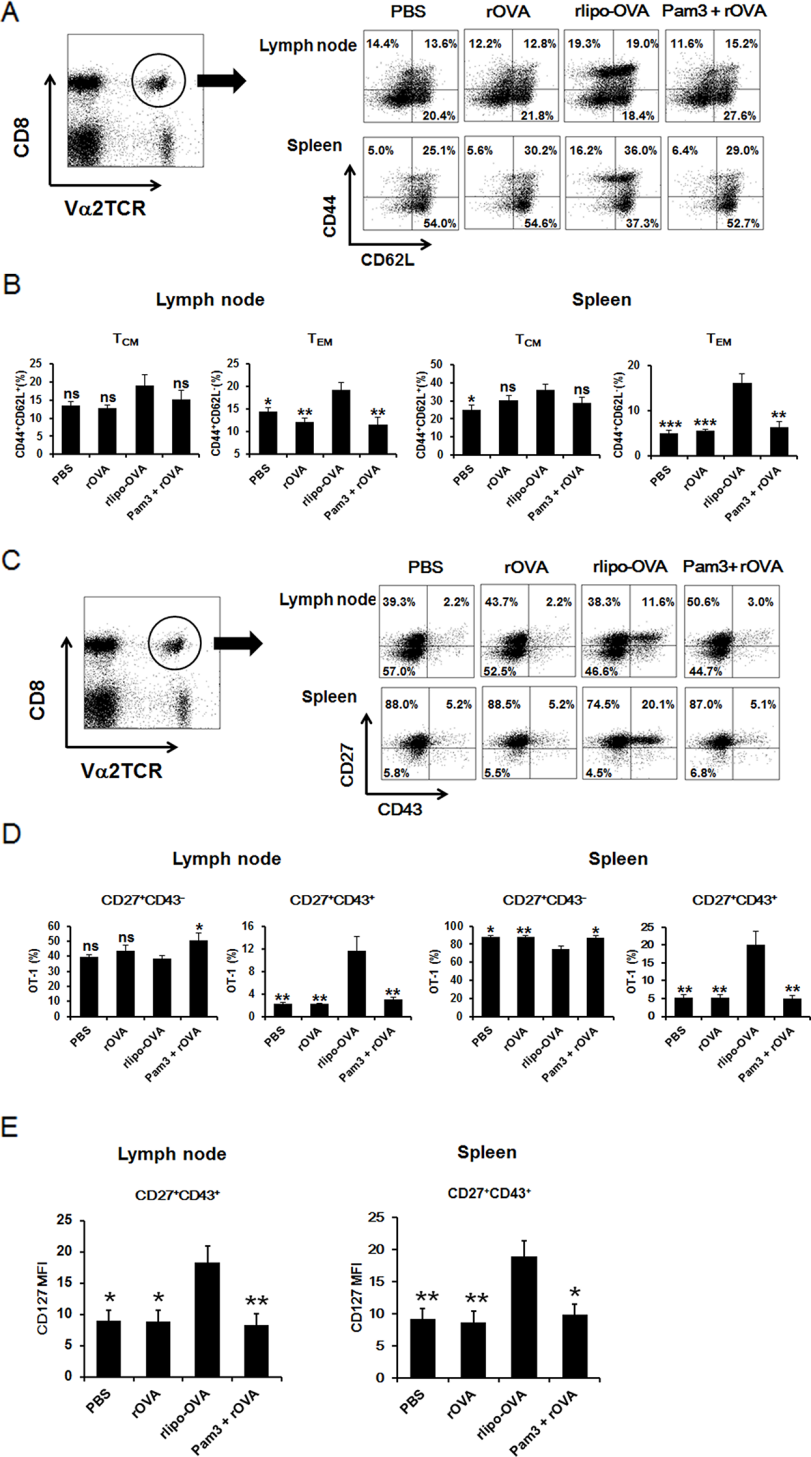
Immunization with rlipo-OVA increases memory CD8 T cells 5 × 10^5^ OT-I CD8^+^ T cells were adoptively transferred via the tail vein one day before once immunization. The mice were injected in each footpad with either PBS alone, rlipo-OVA (5 μg), rOVA (5 μg), or rOVA (5 μg) formulated with an equal molar amount of Pam3CSK4 in 50 ml of PBS. For the analysis of memory populations, the lymphocytes were pooled from the popliteal and inguinal lymph nodes and splenocytes were isolated from the spleens from the groups of immunized mice 28 days after once immunization. The cells were stained with Va2TCR-PE and CD8a-APC. (**A**) The antibodies CD44-FITC and CD62L-APC were used to evaluate T_CM_ cells (CD44^+^CD62L^+^) and T_EM_ cells (CD44^+^CD62L^−^) (**B**). (**C**) The antibodies CD27-FITC and CD43 PE-Cy7 were used to assess effector CD8^+^ T cells in the memory population evaluated for the expression of CD27^+^CD43^−^ and CD27^+^CD43^+^ (**D**). The CD127 marker was used to evaluate the CD27^+^CD43^+^ memory population in the lymph nodes and spleens (**E**). The data are expressed as the mean + SEM. ns = not significant, *p < 0.05, **p < 0.01 and ***p < 0.001 are significant differences compared to rlipo-OVA, *n* = 6.

Because the induction of long-lasting memory CD8^+^ T cells is critical for successful cancer immunotherapy, we analyzed antigen-specific memory CD8^+^ T cells. OT-I cells were adoptively transferred one day before immunization to evaluate whether the lipidated antigen could elicit specific memory CD8 T cell phenotypes. The lymphocytes from the popliteal and inguinal lymph nodes were pooled, and the splenocytes were isolated from the spleens of the immunized mice on day 28. Based on the expression of the CD44 and CD62L molecules on the OT-I cells, the phenotypes of the CD8^+^ T_CM_ and the CD8^+^ T_EM_ populations were analyzed by flow cytometry (Figure 5A). There were no significant differences in the T_CM_ cells (CD44^+^CD62L^+^) populations among the PBS, rOVA, rlipo-OVA and Pam3+rOVA groups. In contrast, the T_EM_ cells (CD44^+^CD62L^−^) in the rlipo-OVA immunized mice were significantly higher compared with the PBS, rOVA or Pam3+rOVA groups in both the lymph nodes and spleens (Figure 5B). In addition to the traditional classification of the T_CM_ and T_EM_ populations, we measured the recall activity of memory T cells using the CD27 and CD43 molecules. The expression of the CD27 and CD43 molecules was analyzed 28 days after immunization (Figure 5C). Surprisingly, the CD27^+^CD43^+^ population was dramatically increased in the rlipo-OVA immunized mice but not in the PBS, rOVA or Pam3+rOVA groups in both the lymph nodes and spleens (Figure 5D). In contrast, there was no significant difference in the CD27^+^CD43^−^ memory T cell population among these groups. Apart the percentage of memory cells, the numbers of memory cells in the draining lymph nodes and spleens are calculated and shown (Figure S2). Additionally, we analyzed whether the memory T cells exhibited long-lived marker by monitoring the level of CD127 molecules in the CD27^+^CD43^+^ population. The CD27^+^CD43^+^ cells expressed high levels of CD127 in the rlipo-OVA immunization (Figure 5E) and the representative cytometry histograms are shown (Figure S3). These results suggested that rlipo-OVA immunization was able to induce the long-lived effector T_EM_ cells.

### Immunization with rlipo-OVA induces CD8-dependent anti-tumor effects and CD127 is functionally required for long term anti-tumor activity

**Figure 6:**
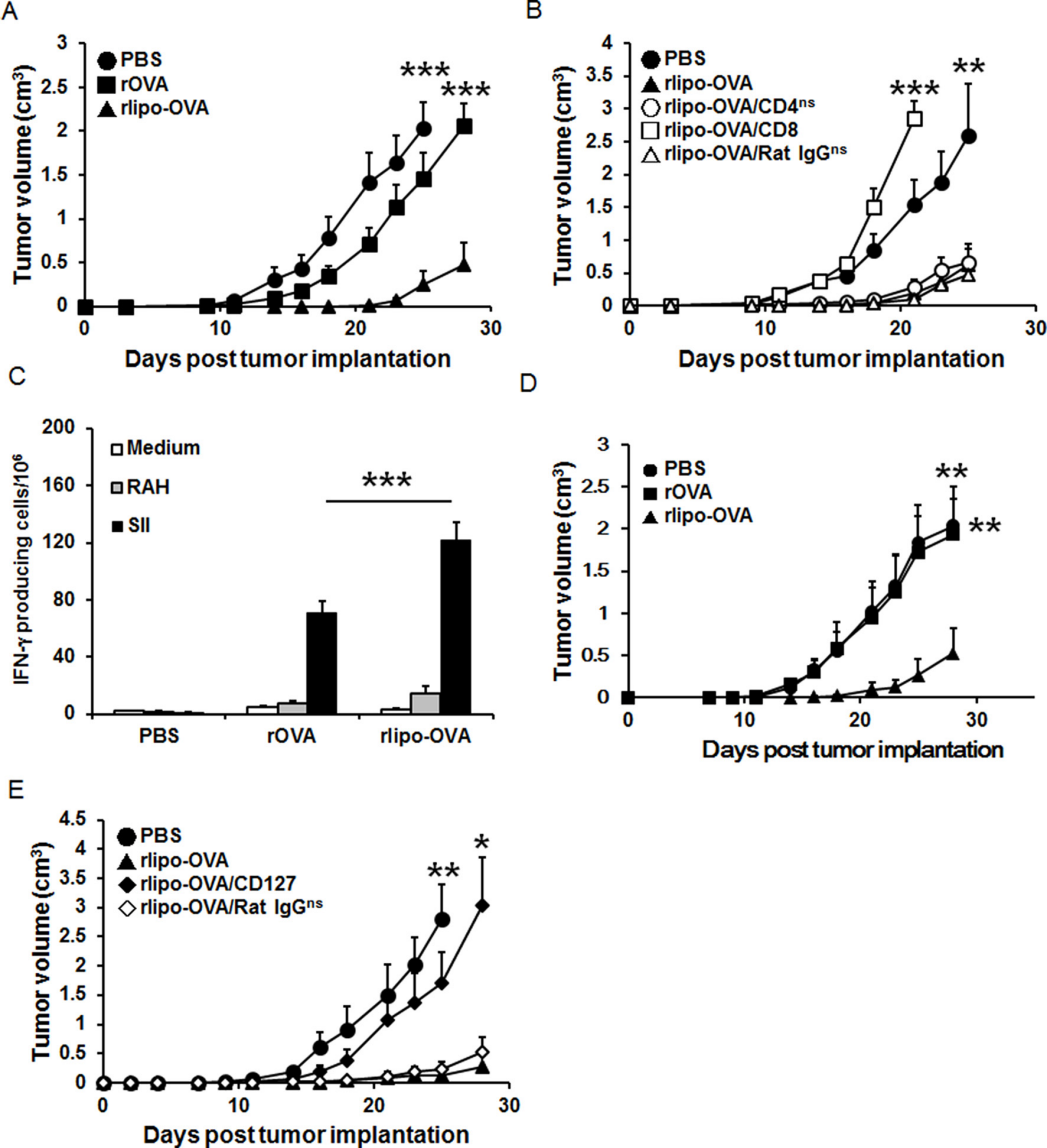
Immunization with rlipo-OVA induces CD8-dependent anti-tumor effects and the functional requirement of CD127 for long term anti-tumor ability (**A**) The mice were inoculated with 2 × 10^4^ EG7 cells in a total volume of 200 μl of PBS. After 3 days, tumor-bearing mice were subcutaneously injected once with rOVA (30 μg), rlipo-OVA (30 μg) or PBS (*n* = 12). (**B**) Three groups (rlipo-OVA/CD4, rlipo-OVA/CD8 and rlipo-OVA/Rat IgG) of mice were injected with anti-CD4, anti-CD8 and control antibodies one day before the injection of rlipo-OVA (30 μg/mouse). On day 3, these groups and two additional control groups (rlipo-OVA and PBS) were subcutaneously injected once with rlipo-OVA (30 μg/mouse) or PBS at the base of tail after inoculation with EG7 cells (2 × 10^4^/per mouse; *n* = 6). (**C** and **D**) Mice were immunized twice by subcutaneous injection of rOVA (30 μg), rlipo-OVA (30 μg) or PBS alone at one-week intervals, and the experiments were performed 4 weeks after the final immunization. (C) The splenocytes (5 × 10^5^ cells/well) from the groups of immunized mice were incubated with or without 5 μg/ml of the RAH peptide or SII peptide for 48 h in an anti-IFN-γ-coated 96-well ELISPOT plate. The IFN-γ-secreting spots were measured using an ELISPOT reader (*n* = 6). (D) The mice were subcutaneously injected with a density of 2 × 10^4^ EG7 cells in a total volume of 200 μl. Tumor growth was observed three times per week (*n* = 8). (**E**) Three groups of C57BL/6 mice were subcutaneously injected with rlipo-OVA (30 μg), and the control group was administered PBS at one-week intervals. On day 17, two groups of immunized rlipo-OVA mice were treated intraperitoneally (i.p.) with either 0.5 mg of rat anti-mouse CD127 antibody or 0.5 mg of rat anti-mouse IgG antibody. A total of 2 × 10^4^ EG7 tumor cells were inoculated on day 35. The data are shown as the means + SE, (*n* = 6). The tumor volume was calculated as the length × width × width × 1/2 (mm^3^). The data are shown as the mean + SEM, ns = no significant difference, *p < 0.05, **p < 0.01 and ***p < 0.001 indicate significant differences compared with rlipo-OVA.

To evaluate whether the rlipo-OVA administration induces anti-tumor immunity, tumor-bearing mice were treated with PBS, rOVA and rlipo-OVA. C57BL/6 mice were subcutaneously injected with 2 × 10^4^ EG7 cells. Three days later, these mice received 30 μg of rlipo-OVA, rOVA or PBS through subcutaneous injection in the dorsum. On day 28 post-tumor implantation, the tumor sizes of the surviving PBS and rOVA-treated mice were greater than 2.0 cm^3^. In contrast, the rlipo-OVA-treated mice were all alive, and their tumor sizes were 0.48 ± 0.25 cm^3^ (Figure 6A). Similar results were obtained when the mice were immunized twice with PBS, rOVA and rlipo-OVA and then subcutaneously injected with EG7 cells 7 days after the final immunization. On day 30 post-tumor challenge, the tumor sizes were barely measurable in the mice that received rlipo-OVA (0.25 ± 0.25 cm^3^), whereas the tumor sizes in the rOVA-treated mice were greater than 2.0 cm^3^ (Figure S4). These results demonstrated that the lipidated immunogen could elicit robust anti-tumor immunity.

To investigate which T cell subset (including CD4 and CD8 cells) contributed to the anti-tumor immunity, we used anti-CD4 (rlipo-OVA/CD4), anti-CD8 (rlipo-OVA/CD8) and control antibodies (rlipo-OVA/rat IgG) to deplete subsets of T cells. The mice were intraperitoneally injected with the antibody one day prior to challenge with EG7 cells. Three days later, the mice were received 30 μg of rlipo-OVA. On day 23 after tumor inoculation, the average tumor sizes in the PBS and rlipo-OVA groups were 1.88 ± 0.48 cm^3^ and 0.36 ± 0.17 cm^3^, respectively. There were no significant differences in tumor sizes among the rlipo-OVA, rlipo-OVA/CD4 and rlipo-OVA/rat IgG groups. In contrast, the protective ability disappeared when the CD8^+^ cells were depleted in the rlipo-OVA/CD8 group (*p* < 0.001, vs. rlipo-OVA; Figure 6B). These data showed that CD8^+^ T cells played a major role in anti-tumor immunity following immunization with rlipo-OVA.

To evaluate whether the induction of memory T cells was important for anti-tumor immunity, the mice were subcutaneously immunized at the base of the tail on days 0 and 7. After 28 days immunization, the splenocytes were harvested to measure the number of OVA-specific IFN-γ-secreting cells using the ELISPOT assay. The results showed that immunization with rlipo-OVA induced higher numbers of SII-specific INF-γ-secreting cells (122.0 ± 12.0) compared with rOVA (70.7 ± 8.6; rOVA vs. rlipo-OVA, *p* < 0.001; Figure 6C). The tumor challenge results were obtained when the mice were immunized twice with immuogens and then subcutaneously injected with EG7 cells 28 days after the final immunization. On day 28 post-tumor challenge, the tumor sizes were measured in the mice that received rlipo-OVA (0.53 ± 0.30 cm^3^), and the tumor sizes in the PBS- or rOVA-treated mice were greater than 2.0 cm^3^ (Figure 6D). These data showed that rlipo-OVA induced long term anti-tumor activity.

To clarify the role of the long-lived effector memory T cells, we used an anti-CD127 antibody to deplete CD127^+^ cells. Ten days after the last immunization, the mice were intraperitoneally injected with anti-CD127 or the control antibody (rat IgG); then, the mice were challenged with EG7 cells on day 35. There were no significant differences between the rlipo-OVA- and rlipo-OVA/rat IgG-treated mice. Interestingly, the protective ability was abolished when the CD127 cells were depleted in the rlipo-OVA/CD127 group (*p* < 0.05, vs. rlipo-OVA, Figure 6E). These data indicated that CD127^+^ T cells were critical for recombinant lipoimmunogen-induced anti-tumor immunity.

## DISCUSSION

Previous studies have explored the important role of memory CD8^+^ T cell differentiation in the rational design of vaccines against virus-infected or cancer cells. Currently, novel vaccine adjuvants are designed to target innate receptors (i.e., TLRs) and to enhance long-lived memory CD8^+^ T cell differentiation. In this report, we investigated whether a TLR2 ligand-fused antigen could increase antigen presentation and generate long-lived memory CD8^+^ T cells. Our data showed that rlipo-OVA could not only activate BM-DC maturation but also efficiently present the SII peptide on MHC I molecules via TLR2 (Figures 2 and 3). After immunization, rlipo-OVA induced higher levels of OVA-specific antibody responses than rOVA (Figure S5A). Consistent with our previous report [33], immunization with rlipo-OVA skewed the immune response towards a Th1-biased immune responses (Figure S5B–S5D). Moreover, rlipo-OVA induced a strong OVA-specific CTL response (Figure 4). The induction of the OVA-specific CTL function capable of inhibiting tumor growth persisted for at least four weeks (Figure 6C and 6D) and the secondary challenge after 80 days immunization also had protection of anti-tumor (Figure S6). Importantly, we found that immunization with rlipo-OVA increased the differentiation of CD44^+^CD62L^−^and CD27^+^CD43^+^ T_EM_ cells; the latter cells expressed high levels of the long-lived CD127 marker. CD127 is also a memory marker, and high expression of the CD127 marker preferentially causes memory cells to persevere and confer immunity for protection [34]. The depletion of CD127^+^ cells led to the loss of the anti-tumor function induced by rlipo-OVA (Figure 6E). These findings suggested that the TLR2 agonist-fused antigen was able to induce long-lived memory CD8^+^ T cells for efficient cancer therapy.

The advantage of the use of a TLR ligand-fused antigen for the induction of CTL responses is the efficient co-localization of the TLR ligands and antigen in the same APCs. Our previous study showed that a TLR2 ligand-conjugated long peptide could regulate the presentation of antigen [15]. Here, we confirmed that peptide/MHC class I complexes were detected on the surface of APCs using OVA as a model antigen (Figure 3). In addition to the TLR2 ligands, the TLR7 agonist conjugated with antigen also enhanced more efficient cross-presentation in DCs and CD8^+^ T cell responses than its unconjugated counterpart [14]. Compared with the free adjuvant, coupling TLR9 ligands to nanoparticles could induce cross-presenting dendritic cells and greatly enhance the adjuvant efficacy at low doses, thereby driving stronger effector CD8^+^ T-cell activation by enhancing their cytolytic profiles and inducing more powerful memory T cells [35]. These data suggested that TLR ligands conjugated to antigen could be efficiently processed and presented to CD8^+^ T cells.

Several TLR ligands can induce memory T cells. Mice vaccinated with a TLR2 agonist-based lipopeptide could induce memory T cells, recall specific T cells, and mediate the reduction of pulmonary viral titers following challenge with virus [36]. The long-lived effector CD8^+^ memory T cells in the memory population are optimal for potent protective immunity due to their longevity and their ability to rapidly expand their numbers and induce protective cytokine and cytotoxic molecules against certain pathogens [34, 37]. TLR4 ligand-elicited memory CD8^+^ T cell differentiation is dependent on MyD88 signals [38]. Interestingly, TLR3 and TLR9 ligands can induce efficient CTL responses and fully functional memory CD8^+^ T cells without CD4^+^ T cell help [39, 40]. Papaya mosaic virus nanoparticles (PapMV) enhanced effector and memory CD8^+^ T cell responses via TLR7 to increase protection against a *Listeria monocytogenes* challenge [41]. Our results showed that once immunization of TLR2 ligand-fused antigen could increase the numbers of CD27^+^CD127^+^ memory T cells (Figure S7). The elimination of CD127^+^ cells failed to induce anti-tumor immunity (Figure 6E). Although CD 27 population is reduced about 60% after prime-boost to compare immune once [42] and the prime-boost regimens are often used to elicit large numbers of memory CD8^+^ T cells [43, 44], but the protection of memory T cell is not decreased by encouraging CD27 negative populations after prime-boost regimens [37]. Moreover, when the immunogen re-challenged, the generated population of CD27^+^CD43^−^ and CD27^+^CD43^+^ have ability to immediately recall killing responses [24]. Here, in Figure 6C–6D and Figure S6, after 28 days prime-boost regimens, the rlipo-OVA could induce anti-tumor ability and also in secondary tumor challenge on day 80. Unexpectedly, we did not observe an increase in T_CM_ cells after rlipo-OVA immunization. This result suggests that T_CM_ differentiation may require a second immunization. The expansion of T_CM_ cells is very rapid after heterologous prime-boosting [45] or viral challenge [46]. Although we did not elucidate the mechanism by which TLR2 regulated the differentiation of T_EM_ or T_CM_ cells, we believe that the induction of cytokine profiles and transcriptomic regulation may contribute to the differentiation of memory T cells (i.e., TLR4 signaling) [38]. However, different TLRs may have different gene expression signatures that may lead to different effects on the differentiation of T_EM_ or T_CM_ cells after vaccination.

Formulating immunogen with proper adjuvants is the most important consideration in developing effective vaccines and conquering the limitations of protein-based immunotherapies. To elicit strong CTLs against cancer cells, the manipulation of the immune response bias to Th1 is critical. Shirota and coworkers [47] reported that the covalent linkage of the antigen with the adjuvant was 100-fold more effective than the unconjugated mixture for Th1 differentiation *in vitro*. In our study, we found that rlipo-OVA induced higher numbers of SII-specific INF-γ-secreting cells than rOVA mixed with the N-terminal segment of recombinant lipoimmunogen (rlipo-Nter; Figure S8). Previous studies have demonstrated the effective adjuvant activities of synthetic lipopeptides, lipoprotein produced from bacteria and lipid-tailed glyco-peptides [48–50]. These results suggested that co-delivery of TLR ligands and antigens could be a promising approach for the development of novel vaccines.

In conclusion, the recombinant lipoimmunogen technology can easily be applied to other tumor-associated antigens with limited constraints. This strategy provides a new direction for the development of successful immunotherapies using protein-based candidates and will hopefully yield safe and effective vaccines for human use.

## MATERIALS AND METHODS

### Chemicals

All experimental chemicals were purchased from Sigma-Aldrich (St. Louis, MO, USA) and Merck (Darmstadt, Germany). Restriction enzymes and the ligase for plasmid construction were purchased from New England Biolabs, Inc. (Beverly, MA, USA). The primers used for cloning were purchased from Mission Biotech, Inc. (Taipei, Taiwan). Trypsin and the matrix for mass spectrometry analysis were purchased from Promega Co. (Madison, WI, USA). Ovalbumin (OVA) grade V (Sigma-Aldrich) used as a control after LPS removal (less than 0.01 EU/μg).

### Cloning and expression of recombinant proteins

The OVA gene of *Gallus gallus* (accession number: P0102) was amplified by conventional PCR. To generate an expression plasmid for rOVA, the following primers were used: forward primer, 5′-GGAATTCCATATGGGCAGCATTGGCGCGGCGAG CAT-3′ (Nde I site underlined) and reverse primer, 5′-CCGCTCGAGCGGGCTCACGCAACGGGCCAAAA AAC-3′ (Xho I site underlined). The PCR product was cloned into the expression vector pET-22b(+) (Novagen, Madison, WI, USA) using the Nde I and Xho I sites to produce the pOVA plasmid. As a result, rOVA contained an additional hexahistidine tag (HisTag) at its C-terminus. The *E. coli* strain BL21 (DE3) (Invitrogen, Carlsbad, CA, USA) was transformed with the expression plasmid pOVA for rOVA expression. The transformed cells were cultured at 20°C overnight in LB broth and then scaled up to 37°C until an OD of 0.6 – 1.0 was reached. rOVA expression was induced by adding 1 mM IPTG at 12°C for 3 days, and the cells were harvested by centrifugation.

Next, we generated the expression plasmid for the recombinant rlipo-OVA. In our previous report [29], pD1E3 was used to generate an rlipo-OVA (pLOVA) expression plasmid. The primers used for this step were as follows: forward primer, 5′-CGGGATCCATGGGCAGCATTGGCGCGGCGAG CAT-3′ (Bam HI site underlined) and reverse primer, 5′-CCGCTCGAGCGGGCTCACGCAACGGCCAAAA AAC-3′ (Xho I site underlined). The PCR product of the OVA gene was cloned into pD1E3 using the Bam HI and Xho I sites to produce the pLOVA plasmid. As a result, the C-terminus of rlipo-OVA contained an additional hexahistidine tag (HisTag). The *E. coli* strain C43(DE3) (Invitrogen) was transformed with the expression plasmid pLOVA for rlipo-OVA expression. The transformed cells were cultured at 20°C overnight in LB broth and then amplified at 37°C until an OD of 0.6 – 1.0 was reached. Protein expression was induced by adding 1 mM IPTG at 12°C for 3 days, and the cells were harvested by centrifugation.

### Purification of recombinant proteins

rOVA was expressed in the *E. coli* BL21(DE3) strain. After the cells were disrupted in a French Press (Constant Systems, Daventry, UK) at 27 Kpsi in homogenization buffer [20 mM Tris (pH 8.0), 50 mM sucrose, 500 mM NaCl and 10% glycerol], the cell lysate of rOVA was clarified by centrifugation (32,000 rpm for 40 min). The supernatant was loaded onto a column (BIO-RAD, Hercules, CA, USA, 2.5 cm i.d. × 10.0 cm) containing 20 ml Ni-NTA resin (Qiagen, San Diego, CA, USA) and washed with the extraction buffer and then the same buffer containing 20 mM imidazole. Then, rOVA was eluted with the homogenization buffer containing 300 mM imidazole. The eluted rOVA was dialyzed to 20 mM Tris (pH 8.0) three times for at least 6 h each time. After dialysis, the rOVA was loaded onto a 20-ml Q Sepharose fast flow column (GE Healthcare, Little Chalfont, Buckinghamshire, UK) and washed with dialysis buffer. The rOVA was eluted with dialysis buffer containing 150 mM NaCl. Mustang E membrane (Pall corporation, NY, USA) was used to remove lipopolysaccharide (LPS) and to exchange the buffer with phosphate buffer saline (PBS). The amount of residual LPS in the rOVA preparations was analyzed using the Limulus amebocyte lysate (LAL) assay (Associates of Cape Cod Inc., East Falmouth, MA, USA). The LPS levels were reduced to less than 5 EU/mg.

To purify rlipo-OVA, the harvested cells were disrupted in a French Press at 27 Kpsi in homogenization buffer. The pellet of the cell lysate was collected by centrifugation (32,000 rpm for 40 min). rlipo-OVA was extracted from the pellet using solubilization buffer [1% Triton X-100 and 20 mM Tris (pH 8.0)]. The extraction supernatant was collected by centrifugation. The supernatant was incubated with 25 ml of Ni-NTA resin (Qiagen) overnight and loaded into a column. The column was washed with the washing buffer [0.1% Triton X-100, 0.3 M NaCl and 20 mM Tris (pH 8.0)] followed by the same buffer containing 30 mM imidazole, and then washed with a 100-fold column volume of 20 mM Tris (pH 7.4) containing 0.1% Triton X-114 to remove the LPS. Next, the column was washed with 20 mM Tris (pH 7.4) to remove the residual detergent, and rlipo-OVA was eluted with 20 mM Tris (pH 7.4) containing 300 mM imidazole. The solubilization buffer was exchanged with PBS. Endotoxin levels were found to be below 0.01 EU/μg.

### Analysis of purified recombinant proteins

The purified rOVA and rlipo-OVA were analyzed by SDS-PAGE, immunoblotting, protein identification and N-terminal amino acid sequencing. The proteins were transferred to a PVDF membrane after separation by SDS-PAGE. The blot was stained with Coomassie Blue R-250. The stained PVDF membrane was washed with de-stain buffer (50% methanol) until bands appeared. The protein bands were excised from the blot. The excised rOVA and rlipo-OVA bands were subjected to four cycles of Edman degradation using an Applied Biosystems Model 494 Protein Sequencer (Mission Biotech). For protein identification, the protein was digested with trypsin for three days at 37°C. The N-terminal fragments in the reaction mixture was further purified using Ziptip (Millipore, MA, USA) after trypsin digestion. The mixture or purified tryptic fragments were mixed with a saturated solution of α-ciano-4-hydroxycinnamic acid in acetonitrile/0.1% trifluoroacetic acid (1:3, vol/vol). The mixture was placed on the target plate of a MALDI-TOF instrument (Waters, Milford, MA, USA) for analysis.

### Splenocyte proliferation assay

Splenocytes from C57BL/6 mice were plated at a density of 2.5 × 10^5^/well in 96-well plates and stimulated with LPS (10, 100, and 1000 ng/ml) or the indicated concentrations of OVA, rOVA, rlipo-OVA or Pam3CSK4 for a total of 72 h at 37°C in a 5% CO_2_ humidified incubator. After 72 h, 10% CellTiter 96^®^ AQ_ueous_ One Solution Reagent (Promega) was added to each well to measure cell proliferation. The absorbance was measured with an ELISA reader at 490 nm. All results are presented as the mean absorbance OD ± standard deviation (SD).

### Activation of BM-DCs

BM-DCs derived from WT, TLR2KO and MyD88KO mice were cultured and assessed as previously described [29]. Briefly, mouse bone marrow cells were cultured at a density of 2 × 10^6^ cells in Petri dishes containing 10 ml of complete RPMI-1640 supplemented with 200 unit/ml (20 ng/ml) recombinant mouse GM-CSF (PeproTech, Rocky Hill, NJ, USA). An additional 10 ml of complete RPMI containing 20 ng/ml GM-CSF was added on day 3. The cells were collected from each dish and counted on day 6. BM-DCs (1 × 10^6^ cells/ml) were stimulated with the indicated concentrations of rOVA, rlipo-OVA, LPS, Pam3CSK4 or medium for 18 h. Cell surface markers [CD11c (clone HL3, BD Biosciences, San Jose, CA, USA), CD40 (clone 3/23, BD Biosciences) and CD80 (clone B7-1, eBioscience, San Diego, CA, USA)] of BM-DCs were analyzed using the FACSCalibur (BD Biosciences). The production of cytokines by BM-DCs (TNF-α and IL-12p40) was determined using ELISA kits (eBioscience).

### Antigen presentation

To investigate OVA-H-2K^b^ production, BM-DCs were incubated for 24 h with PBS, OVA, or rlipo-OVA and stained with CD16/CD32 (clone 2.4G2, BD Biosciences) prior to cell surface staining with the CD11c-FITC (clone N418, Biolegend, San Diego, CA, USA) and SII/H-2K^b^-PE (clone 25-D1.16, eBioscience) antibodies and the isotype control antibody (mouse IgG1κ, BD Biosciences). Then, the cells were subjected to intracellular SII/H-2K^b^ or isotype control antibody staining using the Intracellular Fixation & Permeabilization Buffer Set according to the manufacturer's instructions (eBioscience). To confirm the presentation of SII/H-2K^b^ could activate T cells, BM-DCs (1 × 10^4^) were cultured with 1 × 10^5^ OT-I cells (purity > 90%) which were purified by CD8a^+^ T cell isolation kit (Miltenyi Biotec, Auburn, CA, USA), 1 μCi/well [^3^H]thymidine (Perkin-Elmer Life Science, Boston, MA) was added at 54 h, [^3^H]thymidine incorporation was measured at 72 h of culture, and performing scintillation counting. The level of IFN-γ was determined using ELISA kits (eBioscience) on day 5.

### ELISPOT assay

IFN-γ-secreting cells were calculated using an IFN-γ ELISPOT assay (eBioscience) as previously described [32]. Briefly, splenocytes (5 × 10^5^/well) were added to anti-IFN-γ-coated ELISPOT plates (Millipore) and cultured in medium alone or in the presence of 2.5 μg/ml of the indicated peptides in a final volume of 200 μl of complete RPMI-10. After two days of incubation, the cells were removed by washing the plates with PBST [0.05% (w/v) Tween 20 in PBS]. Then, 10 μg/ml of biotinylated anti-IFN-γ antibody was added to each well in 100-μl aliquots, and the samples were incubated for 2 h. The spots were developed using 3-amine-9-ethyl carbazole (Sigma-Aldrich) and counted by an ELISPOT reader (Cellular Technology Ltd., Shaker Heights, OH, USA).

### Intracellular cytokine staining and flow cytometry analysis

C57BL/6 mice were immunized twice by a subcutaneous injection of rOVA (30 μg), rlipo-OVA (30 μg) or PBS at one-week intervals. Seven days after the final immunization, splenocyte CD8^+^ T cells producing IFN-γ were determined by intracellular cytokine flow cytometry. The peptides used to stimulate the cells were added at a concentration of 2 μg/ml for 18 h at 37°C; then, 1 μl/ml of Brefeldin A (eBioscience), 1 μg/ml ionomycin (Sigma-Aldrich) and 10 μg/ml phorbol 12-myristate 12-acetate (PMA) were added for an additional 4 h before harvesting the cells from the culture. The cells were subjected to intracellular cytokine (IFN-γ) staining using the Intracellular Fixation & Permeabilization Buffer Set (eBioscience) according to the manufacturer's instructions. The cells were washed once with FACS buffer (PBS, 2% FBS and 0.05% sodium azide) and stained with the following monoclonal antibodies: CD16/CD32 (clone 93, eBioscience), CD3ε-PE (clone 145-2c11, Biolegend), CD8a-APC (clone 53–6.7, eBioscience), IFN-γ-PE-Cy7 (clone XMG1.2, eBioscience) and the isotype control antibody (rat IgG1k, eBioscience). Sample acquisition was analyzed using the FACSCalibur (BD Biosciences).

### *In vivo* cytolysis assay

Cytolysis was analyzed using a flow cytometry assay as previously described [51]. Briefly, C57BL/6 mice were immunized twice by subcutaneous injection of rOVA (30 μg), rlipo-OVA (30 μg) or PBS at one-week intervals. Seven days after the final immunization, the splenocytes were washed in PBS. The cells alone (no peptide) and the SII peptide-pulsed cells were labeled at final concentrations of 1 μM CFSE (Molecular Probes, Eugene, OR, USA) and 10 μM CFSE, respectively. The CFSE-labeled cells (2 × 10^7^ cells/mouse) were adoptively transferred via tail vein injection into the immunized mice. The experimental cells from the splenocytes were harvested 18 h after adoptive transfer and analyzed using FACSCalibur. The adjustment factor (A) was calculated by dividing the percentage of cells loaded without peptide by the percentage of cells loaded with SII peptide in the naïve controls. The specific lysis percentage was calculated using the equation:% Specific lysis = [(% non-peptide × A) −% SII peptide] / (% non-peptide × A).

### Phenotype of memory CD8^+^ T cells

C57BL/6 mice received 5 × 10^5^ OT-I CD8^+^ T cells 1 day before inoculation in the footpad with PBS, 5 μg of rlipo-OVA, 5 μg of rOVA, and 5 μg of rOVA formulated with equal molar Pam3CSK4. To evaluate whether the lipidated antigen elicited specific memory CD8 T cell phenotypes, the lymphocytes were pooled from the popliteal, and inguinal lymph nodes and splenocytes were isolated from the spleens from the immunized mice 28 days after immunization. The Fc receptors were blocked with CD16/CD32 (clone 93, eBioscience), and the cells were stained with anti-CD27-FITC (clone LG·7F9, eBioscience), anti-CD44-FITC (clone IM7, BD Bioscience), anti-Va2TCR-PE (clone B20.1, BD Bioscience), anti-CD43 PE-Cy7 (clone 1B1.1, Biolegend), anti-CD127 PerCP-Cy5.5 (clone A71234, eBioscience), anti-CD62L-APC (clone MEL-14, BD Bioscience) and anti-CD8a-APC/Cy7 (clone 53–6.7, BD Bioscience). The acquisitions were determined using the LSRII flow cytometer (BD Bioscience).

### Cell lines and animal studies

EG7 is a mouse leukemia cell line transformed with the ovalbumin plasmid. EG7 cells were cultured in RPMI 1640 (HyClone, Logan, UT, USA) supplemented with 20 mM HEPES (HyClone), 10% heat-inactivated fetal bovine serum (HyClone), penicillin (100 units/ml), streptomycin (100 μg/ml), 1 mM sodium pyruvate, 50 μM β-mercaptoethanol and 0.4 mg/ml G418 at 37°C under 5% CO_2_.

Six- to twelve-week-old female C57BL/6 mice were purchased from the National Laboratory Animal Breeding and Research Center (Taipei, Taiwan). All animals were maintained in accordance with the institutional animal care protocol and housed at the Animal Center of National Health Research Institutes (NHRI, Taiwan). All animal studies were approved by the animal committee of the NHRI for this study (approval ID: NHRI-IACUC-101039-A). In the therapeutic model, the mice were first injected with 2 × 10^4^ EG7 tumor cells in the left flank. On the third day after the tumor challenge, 30 μg of rOVA or rlipo-OVA was administered to the C57BL/6 mice. Tumor diameters were estimated in two orthogonal dimensions using an electronic caliper three times per week. Tumor volumes were calculated with the following formula: (length × width^2^) × 1 / 2.

### Depleting subpopulations of CD4^+^ or CD8^+^ T lymphocytes in mice

The experimental protocols of T cell depletion were modified from a previous study [52]. Briefly, groups of mice were treated intraperitoneally i.p. by injection with 0.5 mg of the rat anti-mouse CD4 antibody (clone GK1.5, eBioscience) and rat anti-mouse CD8 antibody (clone 53–6.72, eBioscience) to deplete CD4^+^ or CD8^+^ T lymphocytes, respectively. A total of 0.5 mg of rat IgG (Invitrogen) was used as a control antibody in experiments. All antibodies were administered on the second day after the inoculation of 2 × 10^4^ EG7 tumor cells. On the third day after the tumor inoculation, 30 μg of rlipo-OVA was subcutaneously injected into the C57BL/6 mice (six per group). The tumor volume was monitored by palpation and inspection.

### Depleting mice of CD127 T lymphocytes in a prophylactic model

The experiments with CD127 T cell depletion have been described previously [34]. Briefly, the C57BL/6 mice were immunized twice by subcutaneous injection of rlipo-OVA (30 μg) and PBS at one-week intervals. On day 17, immunized rlipo-OVA mice were treated i.p. 0.5 mg of rat anti-mouse CD127 antibody (clone SB/14, BD Bioscience) to deplete CD127^+^ T lymphocytes or a rat anti-mouse IgG antibody (clone R35-95, BD Bioscience) as a control. A total of 2 × 10^4^ EG7 tumor cells were inoculated on day 35 (six per group).

### Statistics

All statistical analyses were performed with Prism (GraphPad Software, CA, USA). A two-tailed unpaired Student's *t* test was performed on the data. Ns = no significant difference. Significant differences are represented as follows: *p < 0.05; **p < 0.01; ***p < 0.001.

## SUPPLEMENTARY MATERIALS FIGURES



## Abbreviations

OVA: ovalbumin
rOVA: recombinant ovalbumin
rlipo-OVA: recombinant lipidated ovalbumin
APCs: antigen-presenting cells
CTL: cytotoxic T lymphocyte
T_EM_: effector memory T cells
T_CM_: central memory T cells
