# Supplementary material for: A Toll-like receptor 2 agonist-fused antigen enhanced antitumor immunity by increasing antigen presentation and the CD8 memory T cells population
Source: Oncotarget. 2016 Apr 26;7(21):30804–19. doi: 10.18632/oncotarget.9001 (PMC5058719; doi:10.18632/oncotarget.9001)
Supplement: Supplementary file 1 [file oncotarget-07-30804-s001.pdf]

A toll-like receptor 2 agonist-fused antigen enhanced anti-tumor immunity by increasing antigen presentation and the CD8 memory T cells population

Supplementary Materials

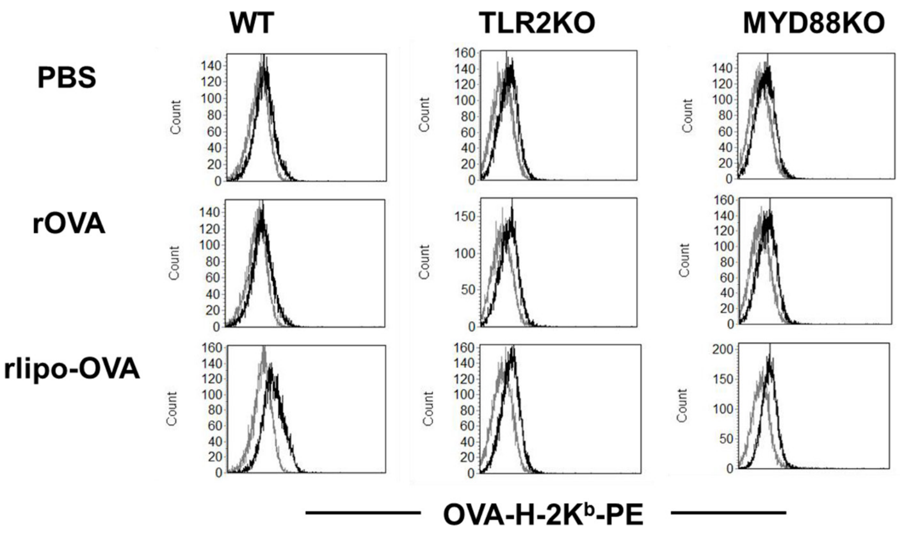

Supplementary Figure S1: The cytometry patterns of rlipo-OVA increases BM-DC presentation of OVA-H-2K^b. BM-DCs from WT, TLR2KO and MyD88KO mice were incubated for 24 h with PBS, 100 nM rOVA, or 100 nM rlipo-OVA. The OVA-H-2K^b of the OVA-peptide was assessed by flow cytometry analysis of cells stained with the PE-labeled 25-D1.16 antibody that recognizes SII assembled with BM-DCs H-2K^b (black line) or the PE-labeled isotype control antibody (gray line).

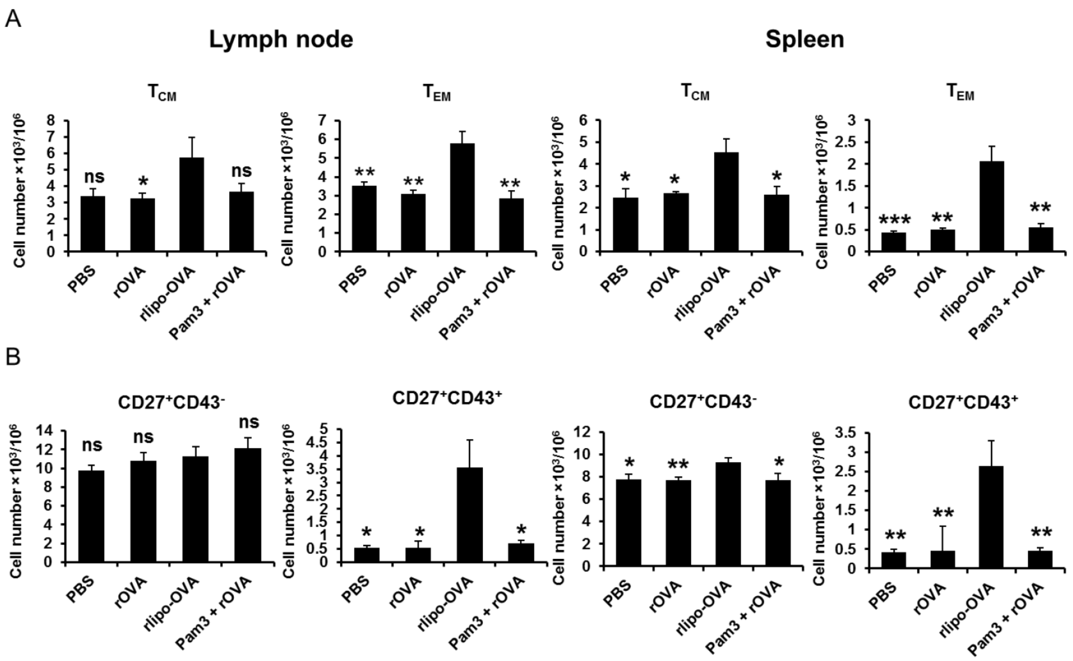

Supplementary Figure S2: The numbers of memory T cells which are induced with rlipo-OVA. The number of memory T cells in 1 million of total cells in Figure 5 are calculated and shown. The data are expressed as the mean + SEM. ns = not significant, **p* < 0.05, ***p* < 0.01 and ****p* < 0.001 are significant differences compared to rlipo-OVA, *n* = 6.

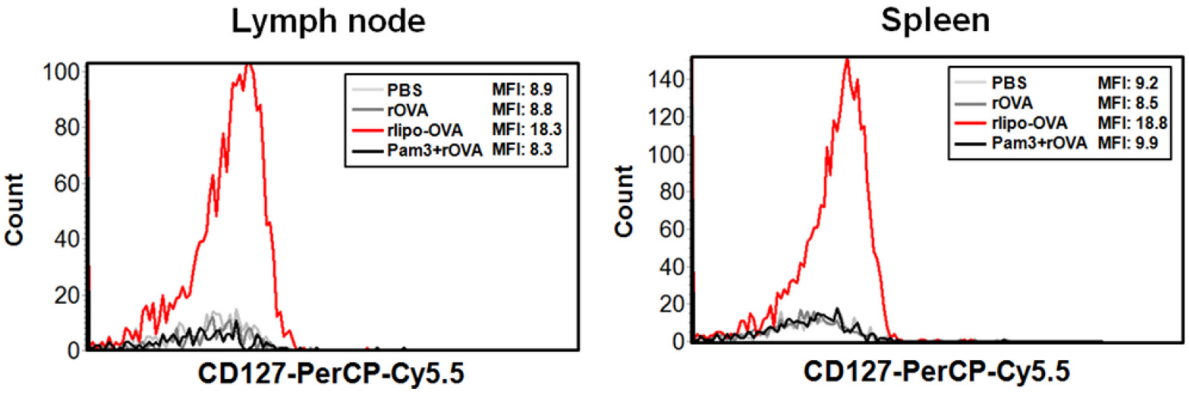

Supplementary Figure S3: The cytometry patterns of rlipo-OVA induces CD127 in the CD27⁺CD43⁺ memory population. The cytometry patterns of CD127 marker in the CD27⁺CD43⁺ memory population in the lymph nodes and spleens are shown.

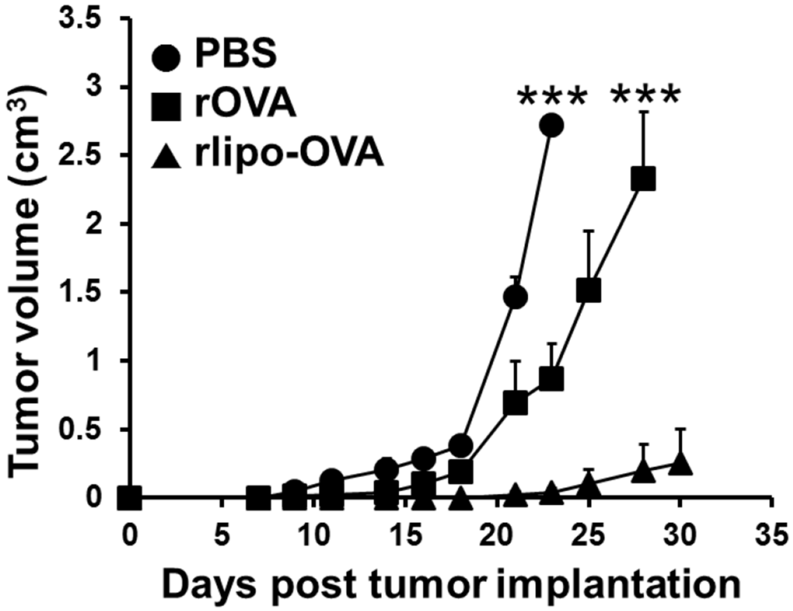

Supplementary Figure S4: rlipo-OVA induces prophylactic anti-tumor ability 7 days after immunization. Mice were immunized two times at the base of the dorsum by either subcutaneous injection of rOVA (10 µg), rlipo-OVA (10 µg) or PBS alone at 7-day intervals. The mice were subcutaneously injected with a density of 2×10^4 EG7 cells seven days after the final immunization. Tumor growth was observed three times per week (*n* = 6; ****p* < 0.001 indicates a significant difference compared with rlipo-OVA).

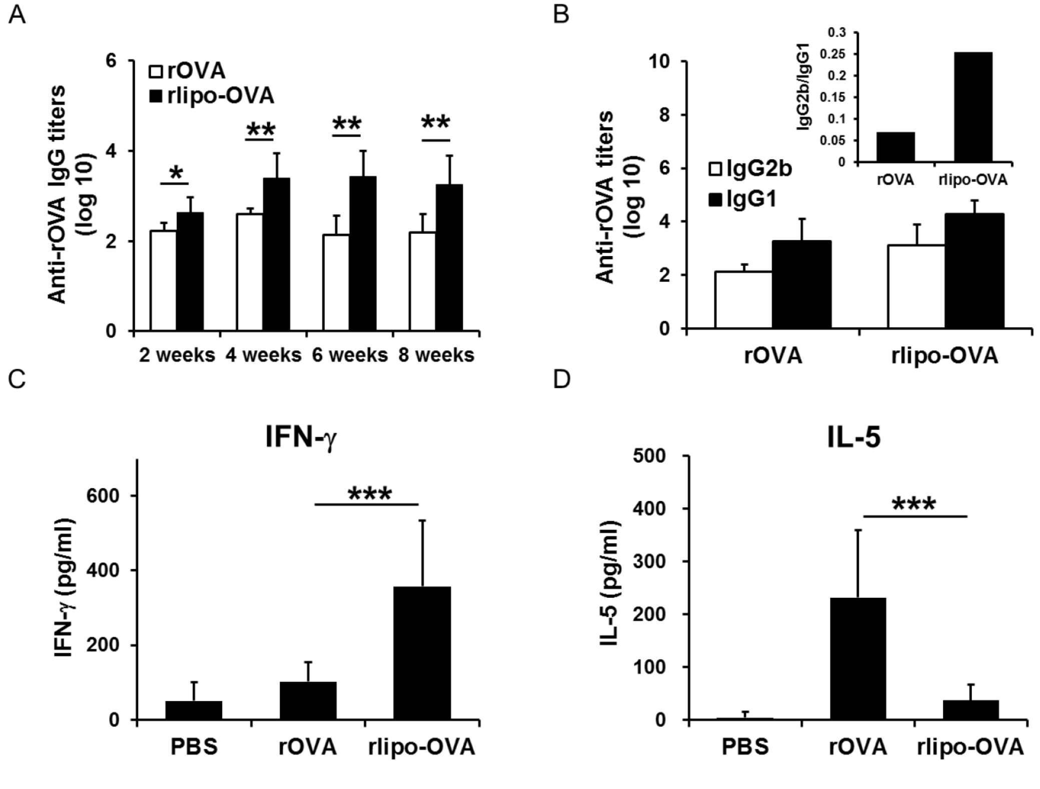

Supplementary Figure S5: Generation of anti-OVA IgG antibody titers and the induction of a Th1-biased response after rlipo-OVA immunization. (**A**) C57BL/6 mice were injected subcutaneously with either 10 µg of rOVA in PBS or 10 µg of rlipo-OVA in PBS twice at two-week intervals. Sera were collected from the groups of mice every two weeks. The horseradish peroxidase (HRP)-conjugated anti-mouse IgG or (**B**) HRP-conjugated anti-mouse IgG2b/HRP-conjugated anti-mouse IgG1 responses against rOVA were assessed by ELISA. The IgG2b/IgG1 ratios in individual groups are shown in the graph. The data are expressed as the mean + SD of the samples (*n* = 6). The mice were injected subcutaneously with 30 µg of rOVA, 30 µg of rlipo-OVA, or PBS alone twice at 7-day intervals. On day 14, splenocytes were isolated and treated with rOVA (10 µg/ml) for 3 or 5 days. The supernatants were collected to assess the Th-bias of the responses. The levels of IFN-γ (**C**) or IL-5 (**D**) were measured by a CBA kit or ELISA, respectively. The results are expressed as the means + SD of the samples (*n* = 6).

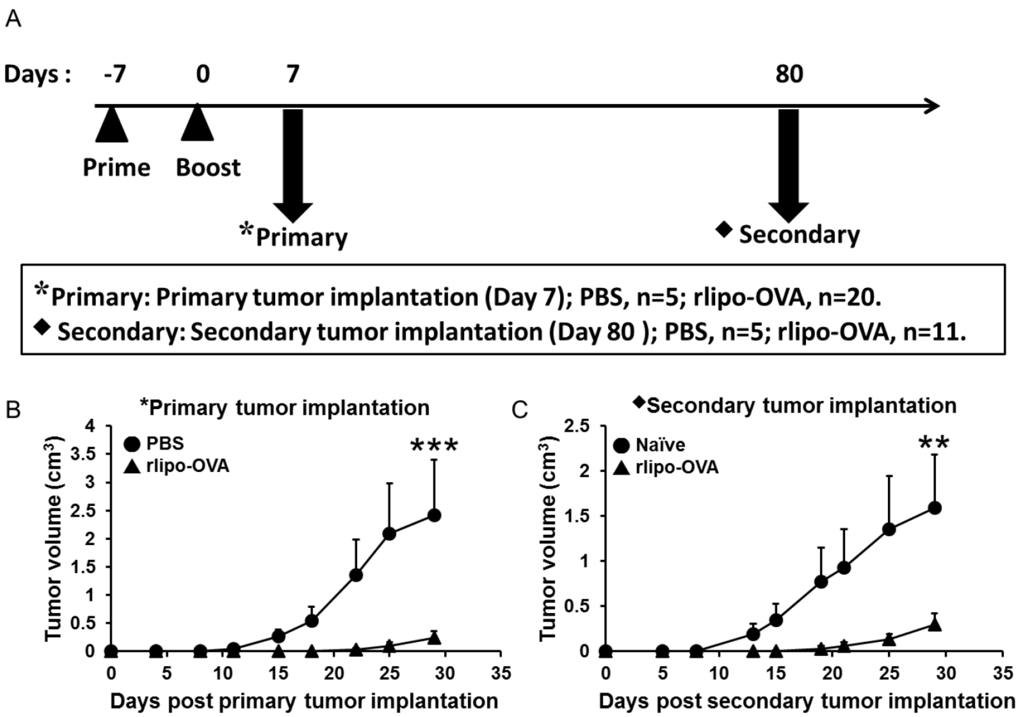

Supplementary Figure S6: rlipo-OVA induces long term prophylactic anti-tumor ability after twice EG7 challenge. (**A**) Schematic protocol for twice EG7 challenge. Mice were immunized twice by subcutaneous injection of rlipo-OVA (30 µg) or PBS alone at one-week intervals. (**B**) At the primary EG7 challenge were performed one week after the final immunization, the mice were subcutaneously injected with a density of 2×10^4 EG7 cells in a total volume of 200 µl. (PBS, $n = 5$; rlipo-OVA, $n = 20$). (**C**) At the secondary EG7 challenge which were performed 80 days after the final immunization, the group of rlipo-OVA was from (A) that without observed any lumps and a group of un-immune mice (Naïve) were as a control. (rlipo-OVA, $n = 11$; Naïve, $n = 5$). Tumor growth was observed three times per week. The data are expressed as the mean + SEM. ***$p < 0.001$ and **$p < 0.01$ indicate significant differences compared with the rlipo-OVA group.

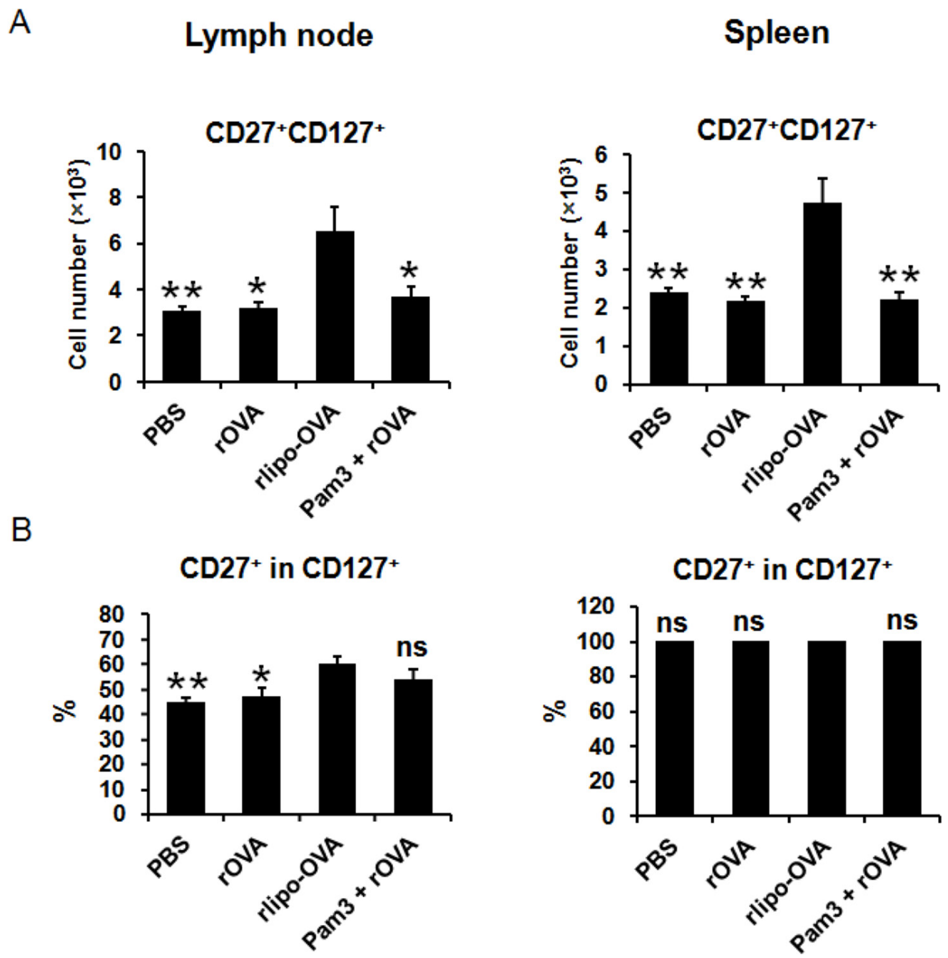

Supplementary Figure S7: rlipo-OVA induces CD27^{+} in the CD127 memory population. (**A**) The number of CD27^{+}CD127^{+} memory T cells in 1 million of total cells and (**B**) the percentage of CD27^{+} in CD127 positive cells in Figure 5 are calculated and shown. The data are expressed as the mean + SEM. ns = not significant, *$p < 0.05$ and **$p < 0.01$ are significant differences compared to rlipo-OVA, $n = 6$.

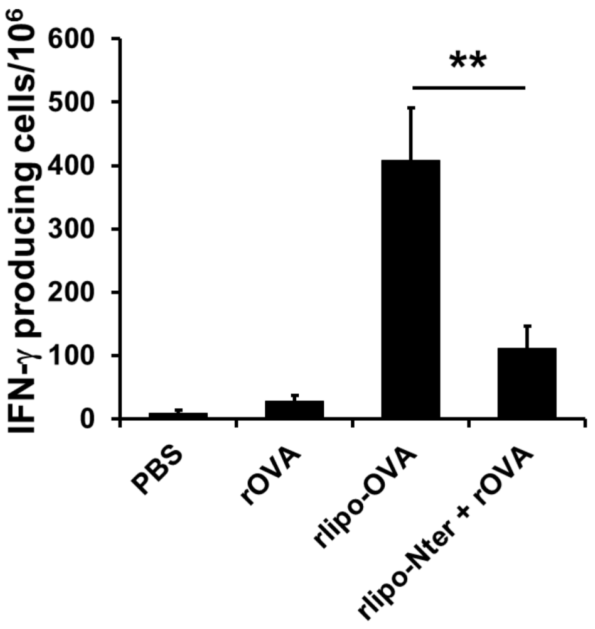

Supplementary Figure S8: rlipo-OVA induced higher numbers of SII-specific INF-γ-secreting cells than rOVA mixed with rlipo-Nter. The mice were immunized twice by subcutaneous injection with PBS alone, rOVA (10 µg), rlipo-OVA (10 µg) or rOVA (10 µg) mixed with equal molar Pam3CSK4 at one-week intervals. At 7 days after the final immunization, the splenocytes (5×10^5 cells/well) from the groups of immunized mice were incubated with 5 µg/ml of the SII peptide for 48 h in an anti-IFN-γ-coated 96-well ELISPOT plate. The IFN-γ-secreting spots were measured using an ELISPOT reader.
